# Association of unmet discharge needs on readmission or mortality among acute respiratory failure survivors during the COVID-19 pandemic: The Addressing Post-Intensive Care Syndrome (APICS-COVID) multicenter prospective cohort study

**DOI:** 10.1371/journal.pone.0361770

**Published:** 2026-11-06

**Authors:** Harris L. Carmichael, Danielle Groat, Victor D. Dinglas, Somnath Bose, Mustafa Mir Kasimov, James C. Jackson, Naga Preethi Kadiri, Maria Isabel Trejo-Zambrano, Carla M. Sevin, Tom Greene, Dale M. Needham, Samuel M. Brown, for the APICS-COVID Study Team

**Affiliations:** 1 Department of Hospital Medicine, Intermountain Medical Center, Murray, Utah, United States of America; 2 Department of Medicine, University of Utah, Salt Lake City, Utah, United States of America; 3 Department of Critical Care Medicine, Intermountain Medical Center, Murray, Utah, United States of America; 4 Outcomes After Critical Illness and Surgery (OACIS) Group, and Pulmonary and Critical Care Medicine, School of Medicine, Johns Hopkins University, Baltimore, Maryland, United States of America; 5 Department of Anesthesia, Critical Care and Pain Medicine, Beth Israel Deaconess Medical Center, Harvard Medical School, Boston, Massachusetts, United States of America; 6 Center for Anesthesia Research Excellence, Department of Anesthesia, Critical Care and Pain Medicine, Beth Israel Deaconess Medical Center, Boston, Massachusetts, United States of America; 7 Division of Pulmonary Medicine, University of Utah, Salt Lake City, Utah, United States of America; 8 Section of Pulmonary and Critical Care Medicine, George E Wahlen VA Medical Center, Salt Lake City, Utah, United States of America; 9 Division of Allergy, Pulmonary, and Critical Care Medicine, Vanderbilt University Medical Center, Nashville, Tennessee, United States of America; UN Mehta Institute of Cardiology and Research Center, INDIA

## Abstract

**Background:**

Survivors of acute respiratory failure (ARF) often face long-term impairments known as Post-Intensive Care Syndrome. These impairments, coupled with fragmented healthcare, contribute to high rates of readmission and mortality. The COVID-19 pandemic challenged healthcare systems, providing an important opportunity to evaluate healthcare fragmentation before and during the pandemic.

**Methods:**

From December 2020 to July 2022, this prospective multicenter study (APICS-COVID) enrolled ARF survivors discharged home from acute care hospitalization. The primary exposure was unmet healthcare needs within 1−4 weeks post-discharge and the primary composite outcome was 3-month readmission and mortality. We additionally compared unmet healthcare needs in this study versus a predominately pre-pandemic cohort from an identical study protocol (APICS-01).

**Results:**

Of 201 patients enrolled, 94% had at least one unmet healthcare need. The median (IQR) number of healthcare needs upon discharge was 18 (11–25) and the median proportion of unmet healthcare needs was 26% (17−36%). Follow-up appointments were the most frequent unmet need. Higher vs. lower unmet healthcare needs were not significantly associated with 3-month readmission or mortality (aRR 0.61, p = 0.22). Compared to the APICS-01 cohort, the frequency of 3-month readmission/mortality was lower (14% vs. 29%, p = 0.26), despite higher unmet needs in the APICS-COVID cohort (26% vs. 16%, p < 0.001).

**Conclusions:**

Across six US academic hospitals during the COVID-19 pandemic, we did not detect a significant association between higher and lower unmet healthcare needs and 3-month readmission or mortality. Compared to APICS-01, unmet follow-up appointments increased but readmission and mortality decreased. Home health services, durable medical equipment, and medications remained robust during pandemic conditions.

## Introduction

Survivors of acute respiratory failure (ARF) often suffer a range of long-lasting physical, cognitive, and/or mental health impairments after hospital discharge, collectively known as Post-Intensive Care Syndrome (PICS) [1–14]. Recovery from ARF frequently requires complex, multi-component care plans that rely on the timely initiation and coordination of needs during the early discharge period. Prior retrospective analyses of claims-based data suggest that incomplete execution of recommended care during this transition period is associated with higher rates of hospital readmission [15–17]. Additionally, ARF survivors frequently experience fragmented healthcare, with many post-discharge healthcare needs being unmet, representing potentially modifiable breakdowns in care delivery that may exacerbate the risk of short-term readmission and mortality [18–21].

At the onset of the COVID-19 pandemic, healthcare systems struggled with an unprecedented increase in the incidence of ARF and disruptions in healthcare delivery throughout the United States (U.S.) that might have impaired the safe return of ARF survivors to the community after discharge [22–27]. Such disruption in effective delivery of healthcare following hospital discharge has the potential to negatively impact the outcomes of ARF survivors. Understanding healthcare needs and the challenges of ARF survivors during pandemic-related public health emergencies is crucial for developing robust, adaptable care models for future pandemics.

Our prospective multicenter cohort study, Addressing Post-Intensive Care Syndrome (APICS-COVID), enrolling both COVID-positive (COVID+) and COVID-negative (COVID-) ARF patients during the pandemic, was designed to fill this knowledge gap by detailing unmet healthcare needs experienced by ARF survivors across six academic hospitals in the U.S. Our primary aim was to enhance our understanding of how pandemic conditions affected unmet healthcare needs of ARF survivors and the effects of these unmet needs on 3-month mortality and readmissions. Our secondary aim was to compare these findings with an immediately preceding patient cohort recruited via an identical study protocol from the same study sites, APICS-01 [18,19] enrolling from January 2019 to August 2020.

## Study design and methods

### Patient eligibility and recruitment

Enrollment began on 12/22/2020 and concluded 7/6/2022, targeting a sample size of 200 patients. This study protocol was identical to our prior APICS-01 study (including identical study sites) and only differed with the additional data collection of COVID-related elements [18,28]. Inclusion and exclusion criteria are presented in Fig 1 with the recruitment timeline. We enrolled patients with ARF (COVID+ and COVID-) being discharged directly home from their ARF-related hospitalization. ARF was defined as ≥24 consecutive hours of (1) high-flow nasal cannula with FIO2 ≥ 0.5 and flow rate ≥ 30 L/min, (2) noninvasive ventilation, or (3) mechanical ventilation via an endotracheal tube, consistent with prior reports [18,21,28]. Patients were excluded if their respiratory support was deemed to be for reasons other than respiratory failure (e.g., exclusively for airway protection), for whom telephone-based follow-up was not feasible (e.g., homelessness, residence outside of U.S.), or those with preexisting cognitive impairment (informed by the Informant Questionnaire on Cognitive Decline in the Elderly) [7,18,29]. Preexisting cognitive impairment was an a priori exclusion criterion of the parent APICS-01 study protocol and was retained for protocol consistency in APICS-COVID. Post-discharge follow-up across both studies relies on patient completion of validated assessments of anxiety and depression (HADS), PTSD (IES-R), cognition (MoCA), social support (MSPSS), functional status (ADL/IADL), and quality of life (EQ-5D), and inclusion of patients with cognitive impairment would have precluded reliable ascertainment of these outcomes, especially given that some of them cannot be validly completed by a proxy respondent (e.g., HADS, IES-R, MoCA).

**Fig 1:**
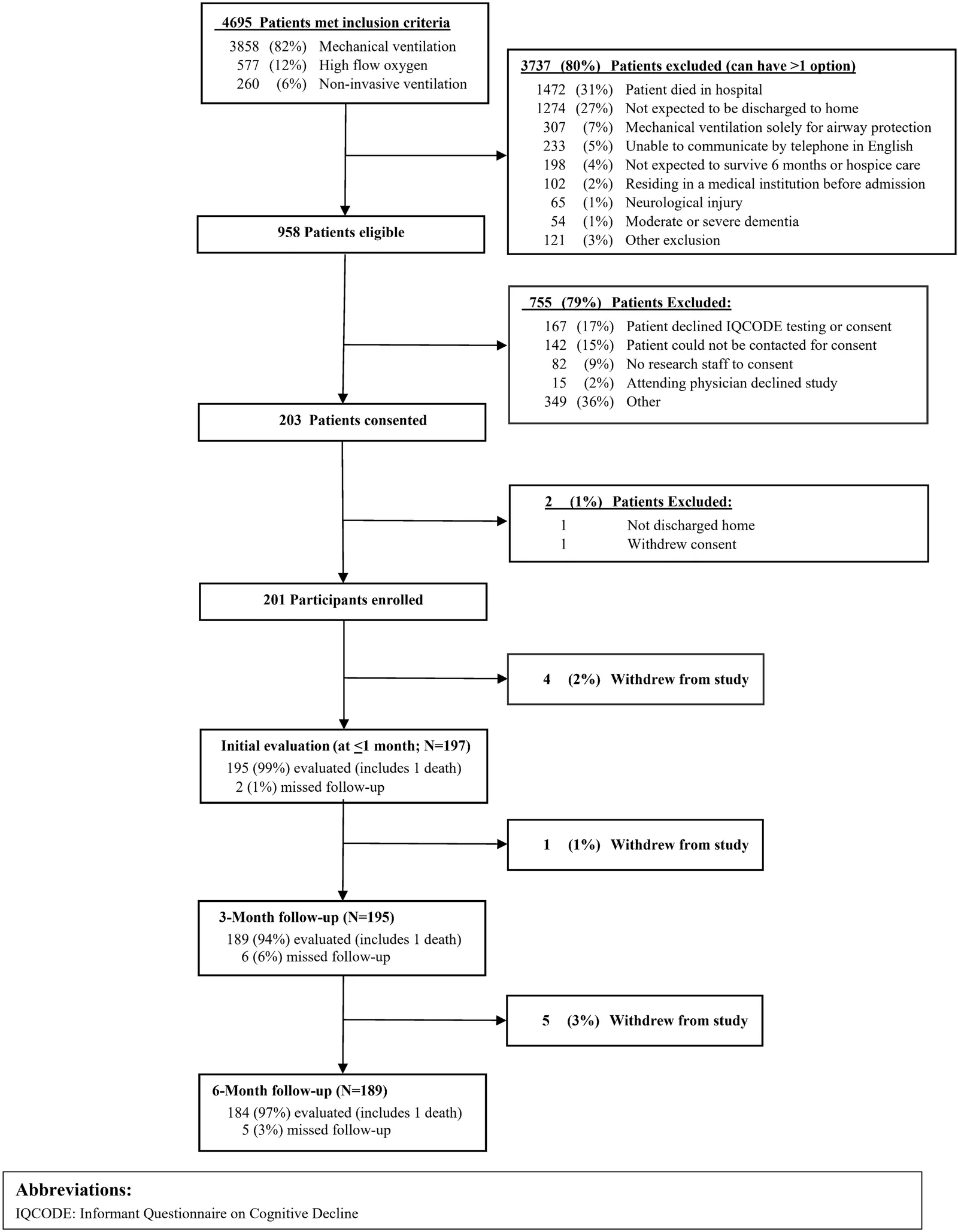
Flow chart depicting patient eligibility, enrollment, and follow-up. Abbreviations: IQCODE: Informant Questionnaire on Cognitive Decline.

### Primary exposure

Unmet healthcare needs after hospital discharge was the exposure of interest. Healthcare needs were established via a previously published structured data collection instrument designed to systematically capture post-discharge needs and their fulfillment for measuring healthcare needs based on hospital discharge documentation and follow-up [28]. The instrument was developed based on recognized needs identified from literature and experience at two ICU aftercare and recovery clinics that were also study site hospitals [30,31]. The instrument was extensively pilot tested across the APICS study sites, with iterative refinement, prior to being finalized for use.

Operational data quality was assured at both stages of data collection. Site-based research coordinators completed the healthcare-needs case report form at hospital discharge while the data coordinating center validated accuracy via virtual site visits for the first patient enrolled at each site and for one randomly selected patient per five subsequent enrollments at each site. Virtual ascertainment of each documented need was then identified as met or unmet by centrally trained callers from the data coordinating center. Each caller completed didactic training, supervised practice, and quality-assurance reviews against a simulated patient and during actual encounters via oversight of an expert interviewer before independent assessment.

Post-discharge needs assessed included both medication-related needs and non-medication needs, such as durable medical equipment (DME, e.g., home oxygen, wheelchair), home health services (e.g., nursing, physical therapy), and follow-up appointments (e.g., primary care or subspecialty provider appointments, community-based rehabilitation appointments not covered under home health). Details regarding the design, pilot testing, and contents of needs assessment instrument are in the e-appendix.

After allowing a 7-day grace period for healthcare needs to be met, their status (met vs. unmet) was determined as soon as possible within 21 days after the grace period. Follow-up appointments were considered met if an appointment had already occurred or was scheduled to occur. Non-medication needs were defined as met if the ordered healthcare need was reported by the respondent as completed. Medication needs were defined as met if a new medication was started, an established medication was discontinued, or a change in dose was implemented as per the hospital discharge plan.

The primary exposure variable was the proportion of unmet healthcare needs, where the denominator was the number of healthcare needs upon discharge, and the numerator was the number of needs unmet in the early discharge period. For example, if a patient had 10 total discharge needs, and 2 were unmet, the proportion of unmet healthcare needs would be 20%. For statistical analysis, the primary exposure was evaluated as higher vs. lower unmet needs based on the median proportion of unmet needs.

### COVID status and outcomes

COVID-19 status was determined based on accepted COVID-19 tests used for clinical purposes at each hospital at the time of the study. The primary outcome was the composite of readmission or death within 3 months of hospital discharge. Secondary outcomes were measured at 3-month and 6-month telephone-based follow-up and included the recommended core outcome set (COS) and associated measurement instruments for ARF survivors reported in the e-Appendix material [32,33]. We have previously reported on the association of COVID-19 status with unmet healthcare needs in this cohort and found no observable effect of COVID-19 status on unmet healthcare needs [34].

### Statistical analysis

The statistical analysis plan was specified before initiating data analysis. Descriptive statistics were reported for each covariate, exposure, and outcome. The primary analysis evaluated the association of unmet healthcare needs on death or readmission within three months of hospital discharge.

A primary consideration for this statistical analysis is controlling for confounding by indication (i.e., more complex discharge plans are made, and the risk for unmet needs is higher, for patients at higher risk for death or readmission at three months). Statistical analysis for this study was conducted in the same manner as the APICS-01 study [18]. In brief, the primary analysis used propensity score methods to estimate the average treatment effect in the treated (ATT). The ATT in this study represents the average difference in the risk of the primary outcome (3-month death or readmission) between patients with high versus low unmet needs, with those observed to have high unmet needs as the treatment group. We used covariate balancing propensity score (CBPS) [35] on the binary exposure to achieve balance on baseline covariates including age, sex, race/ethnicity, insurance, smoking status, and encounter characteristics such as treatments (e.g., invasive support), scores (e.g., APACHE II), and diagnoses (e.g., ARDS [via the Berlin criteria] [36]). Covariate balance was assessed using standardized mean differences and Kolmogorov-Smirnov (KS) statistics for all propensity-model covariates before and after CBPS weighting, with a pre-specified imbalance threshold of 0.15 (S1 Fig).

To estimate the difference in risk and to remain aligned with the marginal cohort balance afforded by the propensity score weighting, we implemented Poisson regression with robust standard errors to estimate the risk ratio, a marginal estimand, and associated confidence interval and p-value of the association between the exposure and outcome [37,38]. Covariates in the final model included age, sex, number of needs, and study site, with propensity scores included as weights. In a post-hoc sensitivity analysis we refit the weighted model using logistic regression with a logit link to estimate the conditional odds ratio, and treated the exposure as a continuous measure (percent unmet needs).

We compared findings from the APICS-COVID study cohort versus APICS-01 study cohort with Fisher’s Exact test for binary parameters and Mann Whitney U test for continuous parameters. All analyses were conducted with R version 4.0.3. Additional methodologic detail and rationale can be found in the e-appendix.

### Ethical considerations

This research was supported by the U.S. Department of Defense through grant number W81XWH-21-1-0050 and was registered on ClinicalTrials.gov before the first participant enrollment (NCT03738774). The research was conducted in accordance with the amended Declaration of Helsinki and received ethical approval and supervision from a centralized Institutional Review Board (IRB) located at Vanderbilt University, with additional oversight provided by the Human Research Protections Office (HRPO) of the Department of Defense. In accordance with local policies, the Veterans Affairs hospital site was subject to the supervision of its designated IRB at the University of Utah. Informed consent was obtained from all participants or their legally authorized representatives. As the study was conducted during the COVID-19 pandemic and was classified as minimal risk, verbal informed consent was permitted in accordance with IRB/HRPO approval. For participants providing verbal consent, a standardized script was read to individuals, and study staff documented agreement using a dedicated consent form. As the study was classified as minimal-risk research an independent third-party witness was not required per federal and local IRB regulations. The standardized oral consent script and the dedicated consent form were both approved by the centralized IRB as components of the study protocol. In cases where consent was obtained prior to hospital discharge, written informed consent was permitted. This manuscript follows the strengthening the reporting of observational studies in epidemiology (STROBE) statement for reporting.

## Results

Of the 4,695 patients meeting inclusion criteria, 958 fulfilled all eligibility criteria, with 203 consented. Among the 203 consented patients, 2 were excluded prior to data collection and 6 withdrew from the study during the initial study procedures, leaving 195/201 (97%) of enrollees achieving follow-up for the primary outcome. We achieved 95% (186/195) follow-up for eligible patients at 6-months. Readmission or death occurred in 27 (13%) and 30 (16%) patients at 3- and 6-months, respectively. These figures include 1 death at each of initial, 3-month, and 6-month follow-up (Fig 1). The median (IQR) time to readmission or death, was 21.5 (12–40) and 117 (68–122) days, respectively.

All patients were discharged with at least 1 medication and 1 non-medication healthcare need, with 184 (94%) having at least 1 healthcare need unmet. The median (interquartile range, IQR) number of healthcare needs at discharge (serving as the denominator for calculating the proportion of unmet healthcare needs) was 18 (11–25). Patient characteristics are reported in Table 1. The median (IQR) age was 54 (42–63) years, 53% female, 42% non-White, and 54% COVID + . Median (IQR) ICU and hospital length of stay was 8 (5–12) and 13 (9–20) days.

**Table 1:** Baseline and hospital encounter characteristics by higher vs. lower unmet discharge needs (n = 195).

Attribute	Lower Unmet Needs, n = 96	Higher Unmet Needs, n = 99	All, n = 195
Age, years, median (IQR)	54 (43-65)	54 (40-62)	54 (42-63)
Female sex, n (%)	51 (53%)	52 (53%)	103 (53%)
Race, n (%)			
Black	24 (25%)	27 (27%)	51 (26%)
Hispanic/Latinx	9 (9.4%)	7 (7.1%)	16 (8.2%)
Non-Hispanic White	59 (61%)	55 (56%)	114 (58%)
Other/multiple	4 (4.2%)	10 (10%)	14 (7.2%)
Body mass index (kg/m2), median (IQR)	32 (28–36)	31(25–40)	32 (26–37)
Respiratory support at enrollment, n (%)			
Invasive mechanical ventilation	45 (47%)	68 (69%)	113 (58%)
Non-invasive mechanical ventilation	9 (9.4%)	4 (4.0%)	13 (6.7%)
High-flow nasal cannula	42 (44%)	27 (27%)	69 (35%)
Transferred from outside hospital, n (%)	16 (17%)	18 (18%)	34 (17%)
APACHE II, score, median (IQR)	16 (12–23)	19 (14–26)	17 (13–24)
Current smoker, n (%)	3 (3.1%)	11 (11%)	14 (7.2%)
Acute Respiratory Distress Syndrome*, n (%)	37 (39%)	59 (60%)	96 (49%)
Clinically Frailty Scale, median (IQR)	3 (2–3)	3 (2–3)	3 (2–3)
Multidimensional Scale Perceived Social Support, median (IQR)	72 (68-83)	71 (60-78)	72 (64-80)
Abnormal AUDIT alcohol use score, n (%)	2 (2.1%)	3 (3.0%)	5 (2.6%)
Charlson Comorbidity Index, median, (IQR)	0 (0-2)	1 (0-2)	1 (0-2)
Hospital length of stay, days. median (IQR)	12 (9–19)	13 (10–20)	13 (9–20)
Resides at home before admission, n (%)	95 (99%)	97 (98%)	192 (98%)
Insurance Status, n (%)			
Private and Public	10 (10%)	9 (9.1%)	19 (9.7%)
Private	51 (53%)	62 (63%)	113 (58%)
Public	29 (30%)	21 (21%)	50 (26%)
None	6 (6.2%)	7 (7.1%)	13 (6.7%)
COVID-19 hospital admission, n (%)	63 (66%)	42 (42%)	105 (54%)
COVID-19 vaccine prior to admission, n (%)	39 (41%)	52 (53%)	91 (47%)
Enrolled in navigator program at discharge, n (%)	2 (2.1%)	2 (2.0%)	4 (2.1%)

Those with ≥26% proportion of needs that were unmet are classified as higher unmet needs and those <26% are classified as lower unmet needs. Abbreviations: IQR: interquartile range; APACHE II: Acute Physiology and Chronic Health Evaluation II; AUDIT: Alcohol Use Disorders Identification Test; COVID-19: COronaVIrus Disease 2019.

*Acute Respiratory Distress Syndrome was diagnosed via the Berlin Criteria [36].

The median proportion of unmet healthcare needs was 26%. Patients with high vs. low unmet needs were of comparable age, sex, race, BMI, functional capacity, and APACHE II score. However, patients with high vs. low unmet needs were less likely to have COVID-19 infection (42% vs. 66%) and more likely to have experienced: ARDS (60% vs. 39%), invasive mechanical ventilation (69% vs. 47%), and COVID-19 vaccinations prior to admission (53% vs. 41%).

Table 2 displays the differences in discharge needs by high vs. low proportion of unmet needs. The median number of discharge medications was 10 for both high and low unmet needs patients. However, those with high vs. low unmet healthcare needs experienced a median of 20% vs. 0% of medication needs as unmet. Patients with high unmet healthcare needs had a median (IQR) of 8 (5–12) non-medication needs, compared with 6 (4–9) non-medication needs for those with low unmet needs. Patients with high unmet healthcare needs had 6 follow-up appointments scheduled, while those with low unmet needs had 4 scheduled. Both groups experienced similarly high rates of unmet follow-up appointments (60% vs 50%). As previously reported, there was no association with COVID-19 status and the rate of unmet discharge needs [34].

**Table 2:** Unmet healthcare needs by type.

Discharge Need	Lower Unmet Needs, n = 96		Higher Unmet Needs, n = 99		All	
	Discharge needs (n)	Unmet, %	Discharge needs (n)	Unmet, %	Discharge needs (n)	Unmet, %
Non-Medication Needs	6.0 (4.0-9.0)	29 (17-43)	8.0 (5.0-12.0)	55 (43-67)	7.0 (4.0-10)	43 (25-57)
Durable Medical Equipment	1.0 (0.0-1.0)	0.0 (0.0-0.0)	1.0 (0.0-1.0)	0.0 (0.0-0.0)	1.0 (0.0-1.0)	0.0 (0.0-0.0)
Home Health Services	2.0 (1.0-3.0)	0.0 (0.0-0.0)	1.0 (0.0-3.0)	0.0 (0.0-33)	1.0 (0.0-3.0)	0.0 (0.0-29)
Follow -Up Appointments	4.0 (2.0-5.0)	50 (24-50)	6.0 (4.0-10.0)	60 (50-75)	4.0 (2.0-7.0)	50 (35-63)
Medications	10 (5.0-16)	0.0 (0.0-6.9)	10 (6.0-15)	20 (0.0-39)	10 (5.0-15)	6.2 (0.0-23)
All Discharge Needs	16 (10-24)	16 (9-20)	19 (14-26)	36 (30-47)	18 (11-25)	26 (17-36)

All data reported as median (interquartile range). Those with ≥26% proportion of needs that were unmet are classified as higher unmet needs and those <26% are classified as lower unmet needs.

Median (IQR) time to readmission was 20 (12-34) days, with 1 death at 19 days (in the high unmet needs group). Readmission rates and time to readmission were similar between those with high vs. low unmet healthcare needs (Table 3). See S1 Table for 3-month and 6-month secondary outcomes by high vs. low unmet healthcare needs.

**Table 3:** Primary outcome and constituents by higher vs. lower unmet healthcare needs.

Outcome	Lower Unmet Needs, n = 96	Higher Unmet Needs, n = 99	All, n = 195
Death or readmission before 3 months, n (%)	15 (16%)	12 (12%)	27 (13.8%)
Time to primary outcome among those achieving primary outcome, days, median, (IQR)	23 (12-38)	20 (13–28)	20 (12-33)
Death before 3 months, n (%)	0 (0.0%)	1 (1.0%)	1 (0.5%)
Time to death among decedent, days, median (IQR)	NA	19 (19 to 19)	19 (19–19)
Hospital readmission before 3 months, n (%)	15 (16%)	11 (11%)	26 (13%)
Time to readmission among those readmitted, days, median (IQR)	23 (12–38)	20 (12–29)	20 (12–34)

Those with ≥26% proportion of needs that were unmet are classified as higher unmet needs and those <26% are classified as lower unmet needs.

Covariates were adequately balanced before final regression models were built (e-Fig 1). High versus low unmet healthcare needs were not associated with the composite outcome of 3-month death or readmission; adjusted risk ratio (aRR) 0.61 (95%CI 0.28–1.33, p = 0.22), with congruent results indicating no association from the sensitivity analysis (S2 Table). None of the 3-month or 6-month secondary outcomes were found to be associated with exposure of unmet healthcare needs (S2 Table).

When comparing the primary exposure and primary outcome between the current pandemic study (APICS-COVID) versus the earlier study (APICS-01), the proportion of unmet health care needs was higher in APICS-COVID (26% vs. 16%, p < 0.001). However, the composite outcome of readmission and/or mortality was lower in APICS-COVID (14%) compared to APICS-01 (29%), but this difference was not statistically significant (p = 0.26).

## Discussion

In this multicenter prospective cohort study of ARF survivors, with and without COVID-19 infection during the pandemic, 94% had at least 1 unmet healthcare need shortly after hospital discharge, with most unmet healthcare needs being non-medication needs. The increase in the proportion of unmet needs between the current study (APICS-COVID) and the identical, immediately preceding study (APICS-01) [18,19] was primarily driven by an increase in unmet follow-up appointments. In contrast, the APICS-COVID vs. APICS-01 study exhibited lower rates of readmission or mortality (primary outcome), although the absolute difference of 15% was not statistically significant. Despite the observed increase in unmet follow-up appointments and the ongoing COVID-19 pandemic, the findings of APICS-COVID were consistent with our prior APICS-01 study: the proportion of unmet healthcare needs did not significantly affect 3-month readmission or death.

We observed a reduction in 3-month readmission or mortality in APICS-COVID as compared to APICS-01 despite identical eligibility criteria, identical study sites, and similarity in some baseline patient characteristics (i.e., female (53 vs. 53%), median patient age (54 vs. 55), Charlson comorbidity index (1 vs 1), clinical frailty scale (3 vs 3), and BMI (32 vs. 30)). However, there were differences between APICS-COVID versus APICS-01 participants in measures of acute illness: median APACHE II score (17 vs. 20), proportion of patients with ARDS (49 vs. 31%), and invasive mechanical ventilation (58 vs. 74%). Notably, study eligibility criteria required discharge to home from the acute care hospital, and pandemic-era changes between the two enrollment windows (e.g., healthcare system strain, altered discharging practices for ARF patients, and evolving clinical management during the COVID-19 pandemic) may have contributed to differences in outcomes observed between the two studies. However, during the timeframe of our study, other reports did not identify significant differences between COVID+ and COVID- ICU patients in terms of inpatient mortality or long-term outcomes [39–41]. Comparative data for post-discharge outcomes in comparable ICU populations could not be found; however, as expected, our readmission rates are higher than those reported for the non-ICU hospitalized COVID-19 population, ranging from 6–9% [42–45].

Home health services and DME were provided with a median of 0% unmet needs in both APICS-COVID and APICS-01. The observed increase in median unmet non-medication healthcare needs was primarily driven by more unmet follow-up appointments in APICS-COVID (50%) compared to APICS-01 (0%). In the APICS-COVID study, we observed that patients with high unmet healthcare needs were less likely to be COVID + ; however, both COVID+ and COVID- patients had the same median unmet follow-up appointment rate of 50%. The median number of follow-up appointments planned at discharge increased to 4 in APICS-COVID, from 3 in APICS-01, with high unmet needs patients, in APICS-COVID study, having a median of 6 planned appointments. This finding may indicate limitations in the medical service delivery during the COVID-19 pandemic [24–27] or may indicate that excessive appointments were planned for APICS-COVID patients, particularly those perceived to be at higher risk of readmission or death during the early phases of a respiratory pandemic. Future research is required to explore differences in patient-level details for unmet appointments in APICS-COVID.

The median (IQR) number of prescribed medication needs was lower in APICS-COVID, 10 (5–15), versus APICS-01, 13 (9–19). The median proportion of unmet medication needs was similar between the two studies at 6%. Hence, comparing the two studies and time periods, there was little difference in the ability of patients to fill discharge medication needs.

Finally, despite APICS-COVID being conducted during the height of the COVID-19 pandemic and observing significantly higher unmet follow-up appointment needs compared with APICS-01, we confirmed our prior finding of no significant effect of unmet healthcare needs on 3-month readmission or mortality for ARF survivors discharging to home. These findings could suggest a phenomenon of over-prescribing to safeguard a population perceived as vulnerable during the COVID-19 pandemic given that there was no effect of unmet needs on readmissions or mortality. However, this suggestion cannot be directly confirmed from the study data. The low proportion of unmet healthcare needs with respect to DME, home health services, and medications from these academic medical centers is consistent with continued availability of these critical services during pandemic conditions.

Several non-mutually exclusive mechanisms could contribute to the lower 3-month readmission and mortality rate observed in APICS-COVID compared with APICS-01 despite the higher proportion of unmet healthcare needs. Pandemic-era care delivery may have produced a healthier subset of patients discharged directly home, consistent with the lower APACHE II scores, lower ARDS prevalence, and lower invasive-ventilation rates observed in APICS-COVID versus APICS-01. This could represent a form of survivor bias that would lower expected readmission and mortality independent of any effect of unmet needs. Increased thresholds for hospital admission during the pandemic may have selectively reduced non-critical readmissions, lowering the observed primary outcome without reflecting genuinely better clinical trajectories among discharged patients. Expanded outpatient and telehealth-based monitoring during the pandemic may also have substituted for some scheduled follow-up appointments (in-person or telehealth enabled), meaning that needs our instrument classified as unmet may have been functionally met through care channels. This would attenuate the operational equivalence of an unmet-need count across the two enrollment windows. These mechanisms are not directly measured in our cohort, but each represents a plausible alternative to the interpretation that unmet healthcare needs are unassociated to outcomes.

The strengths of this study include its multicenter design and the high degree of cohort retention. Limitations include its observational design (albeit robust causal inference analyses were performed), the lack of detailed information regarding the appropriateness of discharge needs, and the unexpected reduction in the rate of our primary outcome compared to available data prior to the study. ARF survivors with preexisting cognitive impairment, excluded at enrollment, constitute a distinct target population whose post-discharge needs and outcomes are not addressed by this analysis and warrant dedicated future investigation. Additionally, the study's sample size limits our power to detect more modest effects of unmet healthcare needs on readmission and mortality. The observed point estimate is consistent with a wide range of associations, and the non-significant result should not be interpreted as evidence of no effect (i.e., type II error cannot be excluded). Although propensity score methods and model covariates were used to reduce confounding by indication, residual confounding from unmeasured factors could still be present. Direct comparisons between APICS-COVID and our preceding APICS-01 cohort should be interpreted as descriptive rather than a formal test of temporal change in the unmet-needs and patient outcome relationship given pandemic-era differences between the two enrollment time windows. Lastly, all study sites were academic centers; hence, study findings may not be generalizable to smaller or lower resourced healthcare systems. Hypothesis generating analyses that investigate the heterogeneity of the exposure and outcome across patient characteristics in an important direction for future work in larger samples.

## Conclusion

In this APICS-COVID study, after accounting for baseline characteristics, severity of illness, and type of respiratory support, we confirm our previous finding from APICS-01 of no significant effect of unmet healthcare needs on 3-month readmission or mortality in our population. Compared to the identical and immediately preceding APICS-01 study (conducted primarily prior to the pandemic), we report that unmet healthcare needs among ARF survivors increased due to more unmet follow-up appointments, while 3-month post-discharge mortality and readmission rates decreased. APICS-COVID also confirms robust delivery of post-discharge DME, home health services, and medication management during a pandemic period that saw strained healthcare resources.

## Supporting information

S1 Text Supplemental methods. (DOCX)

S1 Fig Covariate balance of observed and adjusted parameters based on exposure higher vs. lower unmet healthcare needs. Abbreviations: APACHE II: Acute Physiology and Chronic Health Evaluation II; AUDIT: Alcohol Use Disorders Identification Test; BMI: Body Mass Index; CFS: Clinical Frailty Scale; ARDS: Acute Respiratory Distress Syndrome; COVID-19: COronaVIrus Disease 2019; MSPSS: Multidimensional Scale of Perceived Social Support.
(JPG)

S1 Table Secondary outcomes based on higher vs. lower unmet healthcare needs. Abbreviations: IES-R PTSD: The Impact of Event Scale – Revised, Post-Traumatic Stress Disorder; MoCA: Montreal Cognitive Assessment; MSPSS: Multidimensional Scale of Perceived Social Support; ADL: Activities of daily living; IADL: Instrumental Activities of Daily Living; EQ-5D VAS: EuroQol 5 Dimension Visual Analogue Scale.
(DOCX)

S2 Table Adjusted risk ratio of exposure to above the median proportion unmet healthcare needs and primary composite outcome at 3-months and 3- and 6-month secondary outcomes. Abbreviations: HADS: Hospital Anxiety and Depression Scale; IES-R: The Impact of Event Scale – Revised; MoCA: Montreal Cognitive Assessment; MSPSS: Multidimensional Scale of Perceived Social Support; ADL: Activities of daily living; IADL: Instrumental Activities of Daily Living; EQ-5D VAS: EuroQol 5 Dimension Visual Analogue Scale. *Presented as an Odds Ratio estimated from logistic regression and continuous exposure to percent unmet needs.
(DOCX)

## Abbreviations

APACHE II: Acute Physiology and Chronic Health Evaluation II
APICS Study: Addressing Post-Intensive Care Syndrome Study
AUDIT: Alcohol Use Disorders Identification Test
ARF: Acute Respiratory Failure
COVID-19: COronaVIrus Disease 2019, Severe Acute Respiratory Syndrome CoronaVirus 2 (SARS-CoV-2)
COVID+: Patients with positive result for COVID-19 at enrollment
COVID-: Patients without positive result for COVID-19 at enrollment
IQR: InterQuartile Range
IRB: Institutional Review Board
SOFA: Sequential Organ Failure Assessment Score
STROBE: Strengthening the reporting of observational studies in epidemiology statement
U.S.: United States
