# Supplementary material for: Association of unmet discharge needs on readmission or mortality among acute respiratory failure survivors during the COVID-19 pandemic: The Addressing Post-Intensive Care Syndrome (APICS-COVID) multicenter prospective cohort study
Source: PLoS One. 2026 Nov 6;21(11):e0361770. doi: 10.1371/journal.pone.0361770 (PMC13742942; doi:10.1371/journal.pone.0361770)
Supplement: Supplementary file 1 [file pone.0361770.s001.docx]

# Supporting Information

## Methods:

Sample size was determined a priori based on data from our preceding APICS-01 cohort. Assuming a 24% post-discharge attrition rate, enrollment of 200 patients was predicted to yield 152 patients in the analytic cohort, providing 80% power at two-sided alpha=0.05 to detect an increase in the risk of the composite primary outcome from 30% (in the lower than median proportion of unmet healthcare needs group) to 53% (in the higher than median proportion of unmet healthcare needs group). We achieved n=195 in the analytic cohort.

The instrument for evaluating unmet needs was developed, pilot tested and used for the APICS-01 study. This same instrument was then used for the APICS-COVID study. Full details regarding this instrument was previously published,^25^ as follows:

“In developing the instrument for documenting post-discharge healthcare needs, we initially reviewed published literature and the clinical experience at two participating study sites: the ICU Recovery Center at Vanderbilt University Medical Center and the Aftercare and Recovery Clinic at Intermountain Medical Center. This process was used for “item generation” for the instrument via identifying common post-discharge needs encountered in the literature and in clinical practice. We then conducted content testing of the instrument with the study’s protocol committee, research staff members, and clinicians involved with discharge planning at study site hospitals (e.g., case managers, social workers, home care coordinators; see S1 Table). We also reviewed the draft instrument with the Intermountain Medical Center Patient-Family Advisory Council, incorporating their focused input on language and comprehensibility as well as content. We next pilot tested the instrument, using example discharge documentation, to confirm that instrument covered all relevant post-discharge healthcare needs. Pilot testing (n=20) was conducted until no new items were identified for addition to the instrument.

The resulting instrument contains 13 sections in total of 8 pages and includes items for durable medical equipment, home health services, dialysis, tube feeding, referral to adult protective services, recommendations for inpatient nursing/rehab facilities, and follow-up healthcare-related appointments including physiotherapy, occupational therapy, laboratory tests, imaging, primary care physician, specialist physician, psychologist/psychiatrist, and substance abuse counseling. The other important element of discharge plan is medication optimization, which we also included in the instrument, dividing medications into pre-existing medications that need to be stopped and medications to be started/continued.”
