# Supplementary material for: Association of unmet discharge needs on readmission or mortality among acute respiratory failure survivors during the COVID-19 pandemic: The Addressing Post-Intensive Care Syndrome (APICS-COVID) multicenter prospective cohort study
Source: PLoS One. 2026 Nov 6;21(11):e0361770. doi: 10.1371/journal.pone.0361770 (PMC13742942; doi:10.1371/journal.pone.0361770)
Supplement: Supplementary file 2 [file pone.0361770.s003.docx]

### S1 Table: Secondary outcomes based on higher vs. lower unmet healthcare needs

| Attribute | Lower Unmet Needs | Higher Unmet Needs | All |
| --- | --- | --- | --- |
|  | Median (IQR) | Median (IQR) | Median (IQR) |
| **3-Month Outcomes** |  |  |  |
| HADS Anxiety, median (IQR), n=168 | 3 (1-5) | 3 (1-6) | 3 (1-6) |
| Abnormal >= 8, n (%) | 14 (17%) | 14 (17%) | 28 (17%) |
| HADS Depression, median (IQR), n=168 | 3 (1-4) | 2 (1-6) | 2 (1-6) |
| Abnormal >= 8, n (%) | 10 (12%) | 17 (21%) | 27 (16%) |
| IES-R PTSD, median (IQR), n=165 | 0.5 (0.1-0.7) | 0.5 (0.1-1.1) | 0.5 (0.1-0.9) |
| Abnormal >= 1.6, n (%) | 9 (11%) | 9 (11%) | 18 (11%) |
| MoCA, median (IQR), n=154 | 25 (21-27) | 25 (21-27) | 25 (21-27) |
| Abnormal < 27, n (%) | 56 (70%) | 47 (64%) | 103 (67%) |
| MSPSS, median (IQR), n=162 | 72 (66-74) | 70 (60-72) | 72 (60-73) |
| Abnormal <= 60, n (%) | 16 (20%) | 25 (31%) | 41 (25%) |
| **6-Month Outcomes** |  |  |  |
| ADLs, median (IQR), n=179 | 0 (0-0) | 0 (0-0) | 0 (0-0) |
| Any ADLs, n (%) | 3 (3.4%) | 3 (3.3%) | 6 (3.4%) |
| IADLs, median (IQR), n=179 | 0 (0-1) | 0 (0-0) | 0 (0-0.5) |
| Any IADLs, n (%) | 24 (27%) | 21 (14%) | 45 (25%) |
| EQ-5D VAS, median (IQR), n=170 | 75 (63-90) | 80 (65-90) | 80 (65-90) |
| EQ-5D Utility, median (IQR), n=169 | 0.84 (0.78-1.00) | 0.86 (0.81-1.00) | 0.85 (0.78-1.00) |

Abbreviations: IES-R PTSD: The Impact of Event Scale – Revised, Post-Traumatic Stress Disorder; MoCA: Montreal Cognitive Assessment; MSPSS: Multidimensional Scale of Perceived Social Support; ADL: Activities of daily living; IADL: Instrumental Activities of Daily Living; EQ-5D VAS: EuroQol 5 Dimension Visual Analogue Scale
