# Supplementary material for: Association of unmet discharge needs on readmission or mortality among acute respiratory failure survivors during the COVID-19 pandemic: The Addressing Post-Intensive Care Syndrome (APICS-COVID) multicenter prospective cohort study
Source: PLoS One. 2026 Nov 6;21(11):e0361770. doi: 10.1371/journal.pone.0361770 (PMC13742942; doi:10.1371/journal.pone.0361770)
Supplement: Supplementary file 3 [file pone.0361770.s004.docx]

### S2 Table: Adjusted risk ratio of exposure to above the median proportion unmet healthcare needs and 3- and 6-month secondary outcomes.

| Outcome | Adjusted Risk Ratio (95% CI) | P-value |
| --- | --- | --- |
| **3-Month Outcomes** |  |  |
| HADS Anxiety | 0.874 (0.432 – 1.770) | 0.709 |
| HADS Depression | 2.035 (0.934 – 4.432) | 0.074 |
| IES-R | 0.958 (0.419 - 2.187) | 0.918 |
| MoCA | 0.931 (0.707 - 1.226) | 0.609 |
| MSPSS | 1.164 (0.625 – 2.169) | 0.633 |
| **6-Month Outcomes** |  |  |
| Any ADL impairment | 0.629 (0.140 – 2.822) | 0.545 |
| Any IADL impairment | 0.714 (0.423 – 1.204) | 0.207 |
| EQ-5D VAS | 1.226 (0.076 – 19.75) | 0.886 |
| EQ-5D Utility | 1.284 (0.048 – 34.00) | 0.881 |

Abbreviations: HADS: Hospital Anxiety and Depression Scale; IES-R: The Impact of Event Scale – Revised; MoCA: Montreal Cognitive Assessment; MSPSS: Multidimensional Scale of Perceived Social Support; ADL: Activities of daily living; IADL: Instrumental Activities of Daily Living; EQ-5D VAS: EuroQol 5 Dimension Visual Analogue Scale
