# Supplementary material for: Association of unmet discharge needs on readmission or mortality among acute respiratory failure survivors during the COVID-19 pandemic: The Addressing Post-Intensive Care Syndrome (APICS-COVID) multicenter prospective cohort study
Source: PLoS One. 2026 Nov 6;21(11):e0361770. doi: 10.1371/journal.pone.0361770 (PMC13742942; doi:10.1371/journal.pone.0361770)
Supplement: Supplementary file 4 [file pone.0361770.s006.docx]

**Author Response to revise and resubmit request for manuscript "Effect of unmet discharge needs on readmission or mortality among acute respiratory failure survivors during the COVID-19 pandemic: The Addressing Post-Intensive Care Syndrome (APICS-COVID) multicenter prospective cohort study"
PONE-D-25-58171 - [EMID:e48cf1844384d038]**

Dear Editors,

We are pleased to receive your second revise and resubmit request for our manuscript Addressing Post-Intensive Care Syndrome (APICS-COVID) multicenter prospective cohort study. We appreciate the constructive comments from the reviewers and editors and have addressed their concerns below.

Best Regards,

Harris Carmichael, MD MSHP FACP FHM

**Reviewer #1: The manuscript presents a relevant and timely investigation into post-discharge care fragmentation among survivors of acute respiratory failure during the COVID-19 pandemic. The prospective multicenter design, inclusion of both COVID-positive and COVID-negative patients, and comparison with a pre-pandemic cohort represent important strengths that increase the potential contribution of the study to the field of health services research and critical care survivorship. The topic is clinically meaningful, particularly given the persistent burden of post-intensive care syndrome and the systemic disruptions observed during the pandemic. However, several methodological, analytical, and interpretative aspects require clarification and refinement to strengthen the validity and interpretability of the findings.**

A. **The abstract, while structured and concise, presents conclusions that appear stronger than what the data can fully support. On page 3, the statement that unmet healthcare needs had no effect on outcomes should be tempered, considering the limited sample size and potential residual confounding. The adjusted relative risk of 0.61 with a non-significant p-value suggests uncertainty rather than absence of effect. It would be more appropriate to explicitly acknowledge limited statistical power and the possibility of type II error.**

We agree that our original statement in the results of the abstract implied that there was an absence of an effect from a non-significant result. We have changed the results of the abstract result and conclusion:
*Higher vs. lower unmet healthcare needs were not significantly associated with 3-month readmission or mortality (aRR 0.61, p=0.22).*

*Across six US academic hospitals during the COVID-19 pandemic, we did not detect a significant association between higher and lower unmet healthcare needs and 3-month readmission or mortality.*

the statistical analysis:

*The primary analysis evaluated the effect association of unmet healthcare needs on death or readmission within three months of hospital discharge.*

and results of the main text:

*High versus low unmet healthcare needs were not associated with the composite outcome of 3-month death or readmission; adjusted risk ratio (aRR) 0.61 (95%CI 0.28-1.33, p=0.22), with congruent results indicating no association from the sensitivity analysis. (e-Table 2) None of the 3-month or 6-month secondary outcomes were found to be associated with exposure of unmet healthcare needs. (e-Table 2).*

to help address this important feedback.

Lastly, we expanded our sentence on statistical power in the limitations section with the following:
*Additionally, the study's sample size limits our power to detect more modest effects of unmet healthcare needs on readmission and mortality. The observed point estimate is consistent with a wide range of associations, and the non-significant result should not be interpreted as evidence of no effect (i.e., type II error cannot be excluded).*

B. **The introduction on page 5 provides a solid conceptual background but would benefit from a clearer articulation of the causal hypothesis linking unmet needs to outcomes. While healthcare fragmentation is described, the mechanistic pathways through which unmet needs would lead to readmission or mortality are not sufficiently developed. Expanding this section to include a conceptual framework would enhance coherence between objectives and analysis.**

The first introductory paragraph, has been revised to more explicitly articulate the hypothesized causal pathway linking unmet post-discharge needs to readmission and mortality. The text now describes how PICS necessitates complex care plans during an early vulnerable post-discharge period. We point to prior studies that suggest failures during this transition are associated with early readmissions. We then present fragmented healthcare and unmet needs as potential breakdowns that may increase short-term risk.

*Recovery after critical illness from ARF frequently requires complex, multi-component care plans that rely on the timely initiation and coordination of needs during the early discharge period. Prior retrospective analyses of claims-based data suggest that incomplete execution of recommended care during this transition period is associated with higher rates of hospital readmission.15–17 Additionally, ARF survivors frequently experience fragmented healthcare, with many post-discharge healthcare needs being unmet, representing potentially modifiable breakdowns in care delivery that may exacerbate the risk of short-term readmission and mortality.18–21*

C. **In the methods section, several aspects require further clarification. On page 6, the definition of acute respiratory failure is appropriate, but the exclusion of patients with preexisting cognitive impairment may introduce selection bias by excluding a particularly vulnerable population at high risk of unmet needs and adverse outcomes. The authors should justify this decision more explicitly and discuss its implications for external validity.**

We agree that the basis for the cognitive-impairment exclusion warrants more explicit justification in the main text, and that statements regarding the generalizability of our findings should align with the study population. We have revised the Methods (Patient eligibility and recruitment) to enumerate the validated instruments on which secondary outcomes depend and to make explicit that the pre-existing cognitive impairment was a priori exclusion criterion of the parent APICS-01 protocol which was retained for protocol consistency in this subsequent APICS-COVID study. This parent-study exclusion was based on the fact that tailored care for ARF survivors with baseline cognitive impairment differs substantially from care for ARF survivors without baseline cognitive impairment. We have added a dedicated sentence to the limitations paragraph framing the excluded subgroup as a distinct target population whose post-discharge needs and outcomes are not directly addressed in this analysis.

*Preexisting cognitive impairment was an a priori exclusion criterion of the parent APICS-01 study protocol and was retained for protocol consistency in APICS-COVID. Post-discharge follow-up across both studies relies on patient completion of validated assessments of anxiety and depression (HADS), PTSD (IES-R), cognition (MoCA), social support (MSPSS), functional status (ADL/IADL), and quality of life (EQ-5D), and inclusion of patients with cognitive impairment would have precluded reliable ascertainment of these outcomes, especially given that some of them cannot be validly completed by a proxy respondent (e.g., HADS, IES-R, MoCA).*

*ARF survivors with preexisting cognitive impairment, excluded at enrollment, constitute a distinct target population whose post-discharge needs and outcomes are not addressed by this analysis and warrant dedicated future investigation.*

D. **On pages 6 and 7, the measurement of unmet healthcare needs relies on a customized instrument. Although referenced, the main manuscript lacks sufficient detail regarding its validation, reliability, and reproducibility. A concise summary of its psychometric properties should be included in the main text rather than relegated solely to the appendix.**

We have revised the methods section, starting at line 153, to provide additional detail regarding the development and use of the instrument used to assess post-discharge healthcare needs. The instrument is a structured abstraction tool designed to systematically capture documented post-discharge needs and their fulfillment based on discharge documentation and follow-up. It was developed through a literature-informed process in addition to expert input from investigators who led out-patient follow-up clinics for ICU survivors. It underwent extensive pilot testing to ensure clarity and consistency of abstraction. The instrument has been previously published and used in peer-reviewed prospective studies of PICS (i.e. APICS-01).

Because the instrument identifies discrete, pre-defined healthcare needs (e.g., obtaining wheelchair, follow-up appointment with cardiology) rather than measure an underlying latent construct (e.g., anxiety measured by HADS), traditional psychometric properties such as internal consistency are not the most appropriate framework for evaluation. Additionally, the data coordinating center conducted virtual site visits for independent review of collected patient data for the first patient enrolled at each site and for one randomly selected patient for every five enrollments thereafter by site, totaling duplicate independent review for 50 patients reviewed (25% of enrollees) by study end date. Unmet needs assessment (whether each documented healthcare need was met or unmet) was performed centrally for all sites by trained research staff at the Johns Hopkins University centralized call center. Interviewers at the coordinating center underwent extensive training on how to assess post-discharge needs fulfillment via didactic training and practice sessions, and each was required to pass quality assurance (QA) review using simulated patient and with an actual participant alongside an expert interviewer.

*Unmet healthcare needs after hospital discharge was the exposure of interest. Healthcare needs were established via a previously published structured data collection instrument designed to systematically capture post-discharge needs and their fulfillment for measuring healthcare needs based on hospital discharge documentation and follow-up.28 The instrument was developed based on recognized needs identified from literature and experience at two ICU aftercare and recovery clinics that were also study site hospitals.30,31 The instrument was extensively pilot tested across the APICS study sites, with iterative refinement, prior to being finalized for use.*

*Operational data quality was assured at both stages of data collection. Site-based research staff completed the healthcare-needs case report form at hospital discharge while the coordinating center validated accuracy via virtual site visits for the first patient enrolled at each site and for one randomly selected patient for every five subsequent enrollments at each site. Ascertainment of each documented need was then identified as met or unmet by trained research staff from the centralized call center. Each interviewer completed didactic training, supervised practice, and quality-assurance reviews against a simulated patient and during actual encounters via oversight of an expert interviewer before independent assessment.*

E. **The operationalization of the primary exposure on page 7 raises concerns. Dichotomizing the proportion of unmet needs based on the median may lead to loss of information and reduced statistical power. Modeling the exposure as a continuous variable or exploring alternative categorizations could provide more nuanced insights. Sensitivity analyses using different thresholds would strengthen the robustness of the findings.**

Our a priori analytic plan prespecified dichotomization of the exposure for the primary analysis in order to (1) provide an easy-to-understand estimate, adjusted risk rate, which is interpreted similarly to an odds ratio and (2) not impose assumption of linear or log-linear relationships on the data when planning the analyses. In response to the reviewer’s suggestion, we have added a post-hoc sensitivity analysis treating the exposure as a continuous variable in a propensity-weighted logistic regression. The post-hoc analysis is now described in the methods section, referenced in the results where noted as congruent with the primary analysis, and tabulated in the revised S2 table. We label it as a post-hoc analysis throughout.

*To estimate the difference in risk and to remain aligned with the marginal cohort balance afforded by the propensity score weighting, we implemented Poisson regression with robust standard errors to estimate the risk ratio, a marginal estimand, and associated confidence interval and p-value of the association between the exposure and outcome[37,38]. Covariates in the final model included age, sex, number of needs, and study site, with propensity scores included as weights. In a post-hoc sensitivity analysis we refit the weighted model using logistic regression with a logit link to estimate the conditional odds ratio, and treated the exposure as a continuous measure (percent unmet needs).*

(results section)*…, with congruent results indicating no association from the sensitivity analysis. (e-Table 2)*

F. **The statistical analysis described on page 8 is generally appropriate, particularly the use of covariate balancing propensity scores. However, the description lacks sufficient transparency. The authors should provide more detail on balance diagnostics, including standardized mean differences before and after weighting. Without this information, it is difficult to assess whether confounding was adequately addressed.**

In S1 Fig (previously identified as e-Figure 1) we graphically present the covariate balance diagnostics Kolmogorov-Smirnov (KS) statistics computed before and after CBPS weighting for propensity model covariates based on high vs. low unmet healthcare needs. The high vs. low unmet healthcare needs groups were very similar before covariate balancing with only a few variables falling outside the normal thresholds for mean differences and Kolmogorov-Smirnov and all variables fell within the prespecified threshold after the adjustment. Covariates for balancing were prespecified and consistent with the approach developed in APICS-01. We have expanded the main text methods to describe this pre-specified diagnostic approach.

*Covariate balance was assessed using standardized mean differences and Kolmogorov-Smirnov (KS) statistics for all propensity-model covariates before and after CBPS weighting, with a pre-specified imbalance threshold of 0.15 (S1 fig).*

G. **Additionally, the choice of Poisson regression with robust standard errors is acceptable, but the rationale for this approach over logistic regression should be explicitly justified.**

We have revised the methods and limitations sections to clarify the rationale for using Poisson regression and provided additional references that influenced the analysis plan. We now state that Poisson was selected to estimate the risk ratio, a marginal estimand that aligns with the marginal cohort balance created by the propensity score weighting and the ATT framework. We further clarify that the odds ratio from the logistic regression is a conditional estimand.

*To estimate the difference in risk and to remain aligned with the marginal cohort balance afforded by the propensity score weighting, we implemented Poisson regression with robust standard errors to estimate the risk ratio, a marginal estimand, and associated confidence interval and p-value of the association between the exposure and outcome[37,38]. Covariates in the final model included age, sex, number of needs, and study site, with propensity scores included as weights. In a post-hoc sensitivity analysis we refit the weighted model using logistic regression with a logit link to estimate the conditional odds ratio, and treated the exposure as a continuous measure (percent unmet needs).*

*Although propensity score methods and model covariates were used to reduce confounding by indication, residual confounding from unmeasured factors could still be present.*

H. **The interpretation of the ATT also requires clarification, as the causal language used may overstate the observational nature of the study.**

We have revised the methods section, line 206, to a more accurate interpretation of the ATT. We now indicate that the ATT is an association and an estimate of the difference in the risk of the primary outcome between patients with high vs. low unmet needs, rather than a hypothetical intervention that if implemented would have an effect.

*A primary consideration for this statistical analysis is controlling for confounding by indication (i.e., more complex discharge plans are made, and the risk for unmet needs is higher, for patients at higher risk for death or readmission at three months). Statistical analysis for this study was conducted in the same manner as the APICS-01 study.18 In brief, the primary analysis used propensity score methods to estimate the average treatment effect in the treated (ATT). The ATT in this study represents the average difference in the risk of the primary outcome (3-month death or readmission) between patients with high versus low unmet needs, with those observed to have high unmet needs as the treatment group. We used covariate balancing propensity score (CBPS)35 on the binary exposure to achieve balance on baseline covariates including age, sex, race/ethnicity, insurance, smoking status, and encounter characteristics such as treatments (e.g., invasive support), scores (e.g., APACHE II), and diagnoses (e.g., ARDS [via the Berlin criteria]36).*

I. **On page 9, the comparison with the APICS-01 cohort is potentially informative but methodologically challenging. Although the protocol is described as identical, temporal differences related to the pandemic context introduce multiple uncontrolled confounders, including healthcare system strain, changes in discharge practices, and evolving clinical management. These factors should be more thoroughly acknowledged and discussed as limitations.**

We agree that the possibility of secular confounders raised by the reviewer warrant more detailed discussion from our submission. The discussion limits itself to “changes in practices” and we have expanded this to explicitly name additional pandemic-era factors, including those identified by the reviewer, rather than the abbreviated example as provided. We have added a parallel sentence to the limitations.

*Notably, study eligibility criteria required discharge to home from the acute care hospital, and pandemic-era changes between the two enrollment windows (e.g., healthcare system strain, in the altered discharging practices for ARF patients, and evolving clinical management during the COVID-19 pandemic) may have contributed to differences in outcomes observed between the two studies.*

*Direct comparisons between APICS-COVID and our preceding APICS-01 cohort should be interpreted as descriptive rather than a formal test of temporal change in the unmet-needs and patient outcome relationship given pandemic-era differences between the two enrollment time windows.*

J. **The results section, as presented in the abstract and inferred from the manuscript, would benefit from greater granularity. Specifically, the distribution and types of unmet needs should be more clearly stratified by patient characteristics, COVID status, and site variability. This would help identify subgroups at higher risk and enhance the practical applicability of the findings.**

The reviewer raises three distinct areas of increased granularity (site, COVID-19 status, and other patient characteristics) which we address in turn:

Study site was pre-specified as an adjustment variable in both the covariate-balancing propensity score model and the final model and is controlled for at the analytic stage. Formal stratification inferential models were not performed due to relatively small sample sizes at each given site. This constraint was anticipated in our prespecified analytic plan

Descriptive stratification of the distribution and types of unmet healthcare needs by COVID-19 status in this same APICS-COVID cohort is published in our companion analysis (Carmichael et al., J Hosp Med 2025; reference 29), which examined COVID-19 infection status as the exposure of interest. Tables 1 and 2 of that publication present the COVID-stratified descriptives the reviewer requests, and our methods section cites that analysis. To avoid duplicate publication, we did not replicate these prior analyses within the current paper and kept the current manuscript focused on the primary research question for this paper.

We agree with the reviewer that more granular heterogeneity exploration in population characteristics may be informative for hypothesis generation but are again concerned regarding small sample sizes in these individual subgroups and we have added a sentence to the limitations identifying heterogeneity analyses across patient characteristics as an important direction for future investigation in larger samples.

*Hypothesis generating analyses that investigate the heterogeneity of the exposure and outcome across patient characteristics in an important direction for future work in larger samples.*

K. **The discussion should be revised to better align with the data. The conclusion that certain healthcare components remained “robust” during the pandemic, as stated on page 4, appears speculative without direct measures of system performance. This interpretation should be reframed more cautiously.**

We agree that “robust” as applied to healthcare components overstates the observed data. We have revised this sentence without inferring a systemic claim regarding care delivery.

*The low proportion of unmet healthcare needs with respect to DME, home health services, and medications from these academic medical centers is consistent with continued availability of these critical services during pandemic conditions.*

L. **Furthermore, the manuscript would benefit from a deeper exploration of alternative explanations for the unexpected finding of lower readmission and mortality rates despite higher unmet needs. Possible explanations such as survivor bias, changes in admission thresholds, or increased outpatient monitoring should be considered.**

We agree this point warrants engagement in the discussion. The submitted manuscript briefly noted differences in discharge practices but did not enumerate the broader range of mechanisms. We have added a new Discussion paragraph immediately preceding the limitations articulating the identified three non-mutually-exclusive mechanisms: (1) survivor bias from pandemic-era discharge selection, consistent with the lower APACHE II scores, lower ARDS prevalence, and lower invasive-ventilation rates already reported for APICS-COVID versus APICS-01; (2) elevated hospital admission thresholds during the pandemic that would have selectively reduced non-critical readmissions, consistent with the already-cited pandemic care-delivery literature (references 21–24); and (3) telehealth-based outpatient monitoring substituting for some scheduled in-person follow-up appointments, such that needs our instrument classified as unmet may have been functionally met through care channels the instrument did not capture. The new paragraph notes explicitly that none of these mechanisms is directly measured in our cohort but that each is a plausible alternative to the interpretation that unmet healthcare needs are unrelated to outcomes.

*Several non-mutually exclusive mechanisms could contribute to the lower 3-month readmission and mortality rate observed in APICS-COVID compared with APICS-01 despite the higher proportion of unmet healthcare needs. Pandemic-era care delivery may have produced a healthier subset of patients discharged directly home, consistent with the lower APACHE II scores, lower ARDS prevalence, and lower invasive-ventilation rates observed in APICS-COVID versus APICS-01. This could represent a form of survivor bias that would lower expected readmission and mortality independent of any effect of unmet needs. Increased thresholds for hospital admission during the pandemic may have selectively reduced non-critical readmissions, lowering the observed primary outcome without reflecting genuinely better clinical trajectories among discharged patients. Expanded outpatient and telehealth-based monitoring during the pandemic may also have substituted for some scheduled follow-up appointments (in-person or telehealth enabled), meaning that needs our instrument classified as unmet may have been functionally met through care channels. This would attenuate the operational equivalence of an unmet-need count across the two enrollment windows. These mechanisms are not directly measured in our cohort, but each represents a plausible alternative to the interpretation that unmet healthcare needs are unassociated to outcomes.*

M. **The data availability statement presents a limitation in transparency. While patient privacy concerns are valid, the restriction of data access to requests may not fully comply with open science principles as required by the journal. The authors should clarify whether deidentified datasets or aggregated data could be made more readily accessible.**

Our study protocol, IRB approval, patient consent, and potentially identifiable patient data, preclude our ability to publicly share these data to open data sources without any safety checks. We have presented the data in aggregate format in the current manuscript. De-identified datasets may be obtained through approval from the Intermountain Health Human Research Protections Program at officeofresearch@imail.org as will be stated in the manuscript for publication. This requirement is imposed by Intermountain’s Human Research Protections Program rather than the investigators.

N. **The ethical considerations are adequately described, but the allowance of verbal consent during the pandemic should be briefly justified in terms of potential impact on participant understanding and enrollment bias.**

We thank the reviewer for raising this point. We note that the oral consent procedure was not a pandemic-era allowance. As the submitted Methods (Patient eligibility and recruitment) already notes, "this study protocol was identical to our prior APICS-01 study … and only differed with the additional data collection of COVID-related elements"; the oral consent procedure was an IRB-approved component of that protocol and was used during APICS-01 (which enrolled prior to the COVID-19 pandemic) whenever written in-person consent was not feasible (e.g., a patient discharged before in-person consent could be obtained). The procedure was carried forward into APICS-COVID unchanged. The procedure, standardized script, and the documentation form were unchanged. With respect to participant understanding, all verbal consents followed the IRB-approved standardized oral consent script, ensuring uniform information delivery across sites and study personnel. Trained study staff documented participants’ understanding of the protocol and verbal agreement contemporaneously on a dedicated consent form and logged the consent in the study's data capture system (REDCap).

With respect to enrollment bias, we hypothesize that the availability of an oral consent option reduced, rather than increased, selection bias relative to a written-only approach (i.e., patients who would otherwise have been excluded by visitor restrictions, rapid discharge, or transportation barriers were eligible for enrollment on the same terms as patients consented in person).

**In summary, the manuscript addresses an important question with a solid study design, but requires substantial clarification in its methodological description, more cautious interpretation of findings, and deeper engagement with potential biases and limitations. Addressing these issues will significantly enhance the rigor and credibility of the work.**

**The following questions may help the authors further strengthen the manuscript:
1.How was the sample size determined, and was the study adequately powered to detect clinically meaningful differences in the primary outcome?**

In the e-Appendix we include additional methods and statistical analysis information, including a section specifically for sample size and power calculations.

*Sample size was determined a priori based on data from our preceding APICS-01 cohort. Assuming a 24% post-discharge attrition rate, enrollment of 200 patients was predicted to yield 152 patients in the analytic cohort, providing 80% power at two-sided alpha=0.05 to detect an increase in the risk of the composite primary outcome from 30% (in the lower than median proportion of unmet healthcare needs group) to 53% (in the higher than median proportion of unmet healthcare needs group). We achieved n=195 in the analytic cohort.*

While there are no published norms on the minimum clinically important difference for the composite outcome used in APICS; it is true that an intervention that decreased the rate of admission or death by an absolute 10% could be clinically important. We emphasize in the manuscript that we were not powered to detect a small or moderate effect.

**2.Can the authors provide balance diagnostics for the propensity score model to demonstrate adequate control of confounding?**

See section F above: In brief, the e-Appendix we included as S1 Fig (e-Figure 1) illustrates the covariate balance before and after adjustment.

**3.What are the psychometric properties of the instrument used to assess healthcare needs, and has it been externally validated?**

We included more information on the development of the instrument in the eAppendix that was previously published with the APICS-01 study. For methodological reasons addressing formal psychometric evaluation, see section D above

**4.Why was the primary exposure dichotomized rather than analyzed as a continuous variable?**

As per the discussion above in reviewer comment E: The exposure was dichotomized to facilitate a clear and interpretable comparison between groups with high versus low unmet needs, consistent with the ATT framework used in the primary analysis. This approach avoids imposing strong assumptions about a linear relationship on the log scale between the proportion of unmet needs and the outcome. In short, dichotomization avoids assumptions about the data and preserves interpretability.

**5.Were sensitivity analyses performed using alternative exposure definitions or modeling approaches?**

We have now included a post-hoc sensitivity analysis, as described in E above.

**6.How might the exclusion of patients with cognitive impairment have influenced the study findings?**

Please see the response to review comment C above

**7.Were there any site-level differences in unmet needs or outcomes, and how were these accounted for analytically?**

As addressed in section J: We included study site as an adjustment variable in the final models. All sites were well represented, but at this point we have not done extensive heterogeneity analyses as they were not pre-specified in the analysis plan.

**8.Could differences in healthcare system strain during the pandemic explain the observed reduction in readmission and mortality?**

See section L above

**9.How do the authors address the possibility of residual confounding given the observational design?**

Residual confounding is an inherent concern in observational studies and cannot be completely eliminated. To mitigate confounding by indication, we implemented a doubly robust analytic approach that combines propensity score weighting with outcome regression, providing protection against model misspecification if at least one model is correctly specified. We balanced on a large set of clinically relevant baseline covariates prior to outcome modeling. Additionally, we included the number of needs, or the denominator of the exposure, as an adjustment in the final model, to further reduce the potential structural confounding related to the exposure scale.

**10.Are there plans to make deidentified data publicly available in a repository to enhance transparency and reproducibility?**

See section M above.

**Editor’s Comments:**

**I concur with these comments by reviewer 1. "The operationalization of the primary exposure on page 7 raises concerns. Dichotomizing the proportion of unmet needs based on the median may lead to loss of information and reduced statistical power. Modeling the exposure as a continuous variable or exploring alternative categorizations could provide more nuanced insights. Sensitivity analyses using different thresholds would strengthen the robustness of the findings. The statistical analysis described on page 8 is generally appropriate, particularly the use of covariate balancing propensity scores. However, the description lacks sufficient transparency. The authors should provide more detail on balance diagnostics, including standardized mean differences before and after weighting. Without this information, it is difficult to assess whether confounding was adequately addressed.
Additionally, the choice of Poisson regression with robust standard errors is acceptable, but the rationale for this approach over logistic regression should be explicitly justified. The interpretation of the ATT also requires clarification, as the causal language used may overstate the observational nature of the study. On page 9, the comparison with the APICS-01 cohort is potentially informative but methodologically challenging. Although the protocol is described as identical, temporal differences related to the pandemic context introduce multiple uncontrolled confounders, including healthcare system strain, changes in discharge practices, and evolving clinical management. These factors should be more thoroughly acknowledged and discussed as limitations.
The results section, as presented in the abstract and inferred from the manuscript, would benefit from greater granularity. Specifically, the distribution and types of unmet needs should be more clearly stratified by patient characteristics, COVID status, and site variability. This would help identify subgroups at higher risk and enhance the practical applicability of the findings. The discussion should be revised to better align with the data. The conclusion that certain healthcare components remained “robust” during the pandemic, as stated on page 4, appears speculative without direct measures of system performance. This interpretation should be reframed more cautiously.
Furthermore, the manuscript would benefit from a deeper exploration of alternative explanations for the unexpected finding of lower readmission and mortality rates despite higher unmet needs. Possible explanations such as survivor bias, changes in admission thresholds, or increased outpatient monitoring should be considered. The data availability statement presents a limitation in transparency. While patient privacy concerns are valid, the restriction of data access to requests may not fully comply with open science principles as required by the journal. The authors should clarify whether deidentified datasets or aggregated data could be made more readily accessible."**

Please find our responses to the reviewer comments in question above.

Journal requirement edits:

J1: **Please ensure that your manuscript meets PLOS ONE's style requirements, including those for file naming. The PLOS ONE style templates can be found at**

Please find our formatting changes in the documents

J2: **In the ethics statement in the Methods, you have specified that verbal consent was obtained. Please provide additional details regarding how this consent was documented and witnessed, and state whether this was approved by the IRB.**

The submitted Methods (Ethical considerations) notes that verbal consent was given "in accordance with IRB/HRPO approval" using a standardized script and a dedicated consent form. The standardized oral consent script and the dedicated consent form were both approved by the centralized Institutional Review Board at Vanderbilt University Medical Center as components of the study protocol. As the submitted Methods (Patient eligibility and recruitment) notes, this protocol was carried forward essentially unchanged from the preceding APICS-01 study, including its oral consent provision. The study was classified as minimal-risk research. Under the IRB-approved documentation procedure and applicable federal regulations governing informed consent in minimal-risk research, an independent third-party witness was not required; trained study personnel obtained consent. Study personnel read the IRB-approved standardized oral consent script, documented the participant's (or legally authorized representatives) verbal agreement contemporaneously on a dedicated consent form, and logged the consent in the study's electronic data capture system (REDCap).

We have expanded both the Methods and the Ethical approval section to make the IRB approval, documentation procedure, and witnessing question explicit:

*As the study was classified as minimal-risk research an independent third-party witness was not required per federal and local IRB regulations. The standardized oral consent script and the dedicated consent form were both approved by the centralized IRB as components of the study protocol.*

J3 **Please provide an amended Funding Statement declaring this commercial affiliation, as well as a statement regarding the Role of Funders in your study.**

We have amended the statements to clarify the role of the funder (US Department of Defense) and to clarify there was no role of any commercial affiliations from the conflicts of interest in this study and added to the text:
*The Department of Defense did not have any additional role in the study design, data collection and analysis, decision to publish, or preparation of the manuscript.*

*No commercial interest played any role in the study design, data collection and analysis, decision to publish, preparation of the manuscript, and did not alter our adherence to PLOS ONE policies on sharing data and materials.*

Your message notes that "one or more of the authors are employed by a commercial company." We do not believe this applies to any author on this manuscript. All authors are employed by academic institutions or a not-for-profit health system.

We believe the flag may have been triggered by language describing Dr. Needham's interests related to a separate, completed NIH-funded trial (R01HL132887). In connection with that trial, Dr. Needham was previously in receipt of an unrestricted research grant and donated product from Baxter Healthcare Corporation and an equipment loan from Reck Medical Devices/Enable ME LLC. Dr. Needham is not employed by either entity, and neither provided funding or materials for the present study which is updated in the COI and funding statements to reflect this concern. Similarly, Dr. Brown receives licensing fees from ReddyPort for an airway invention owned by Intermountain Health; he is not employed by ReddyPort but rather this academic affiliation and this has also been updated to reflect this concern in the COI and funding statement.

J4 **If ethical or legal restrictions apply, explain them in detail (e.g., potentially identifying patient information, third-party ownership) and identify the body imposing them (IRB, ethics committee, etc.). Provide contact information for a data-access committee or institutional body to which requests may be sent.**

Our study protocol, IRB approval (Vanderbilt University Medical Center, with additional oversight by the Human Research Protections Office of the Department of Defense), and patient consent preclude our ability to publicly share these data, which contain potentially identifying information from a multi-center prospective cohort. The data have been presented in aggregate format in the manuscript and supporting information. Requests for de-identified data from qualified researchers with appropriate ethics-board approvals and data use agreements may be directed to the Intermountain Office of Research at officeofresearch@imail.org.

J5 **One of the noted authors is a group or consortium [APICS-COVID Study Team]. In addition to naming the author group, please list the individual authors and affiliations within this group in the acknowledgments section of your manuscript. Please also indicate clearly a lead author for this group along with a contact email address.**

Please find the Acknowledgements to additional APICS-COVID Study Team members section of Declarations in the manuscript and the addition of:

*APICS consortium lead and point of contact*

*Samuel M. Brown, MD, MS (SMB: Intermountain Medical Center; Samuel.Brown@imail.org).*

J6 **Please include captions for your Supporting Information files at the end of your manuscript, and update any in-text citations to match accordingly. Please see our Supporting Information guidelines for more information:**

Please find updated supporting information files

J7 **We notice that your supplementary [figures/tables] are included in the manuscript file. Please remove them and upload them with the file type 'Supporting Information'. Please ensure that each Supporting Information file has a legend listed in the manuscript after the references list.**

Please find updated S Fig / Tables files and main manuscript Supporting information section

J8 **If the reviewer comments include a recommendation to cite specific previously published works, please review and evaluate these publications to determine whether they are relevant and should be cited. There is no requirement to cite these works unless the editor has indicated otherwise.**

R1 did not recommend specific citations. Thank you.
